# Mutations in the *Physcomitrium patens* gene encoding Aminodeoxychorismate Synthase confer auxotrophic phenotypes

**DOI:** 10.17912/micropub.biology.000364

**Published:** 2021-01-26

**Authors:** Michael J Prigge, Yingluo Wang, Mark Estelle

**Affiliations:** 1 Section of Cell and Developmental Biology, University of California San Diego, La Jolla, CA 92093-0116, USA

## Abstract

To facilitate genetic mapping of developmental mutants of *Physcomitrium patens*, we produced a genetic marker that combines recessive auxotrophy with dominant positive selection. We first identified the gene affected by the *pabB4* auxotrophic mutation and then replaced it with a cassette that confers antibiotic resistance. This strain may be used to produce bi-parental somatic hybrids with nearly any other strain.

**Figure 1:**
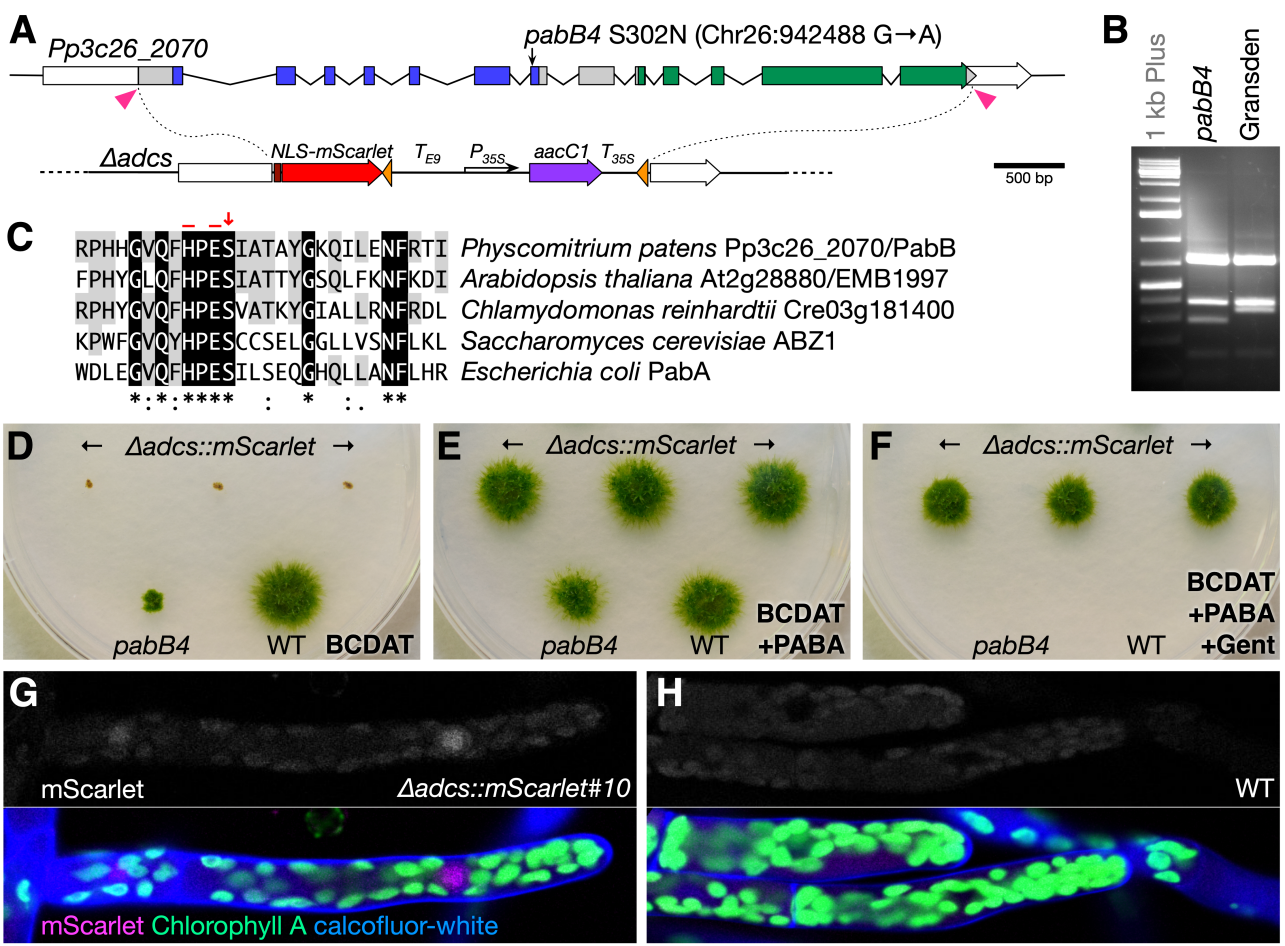
**(A)** Diagram of the *Pp3c26_2070* locus encoding the ADCSenzyme and the gene-replacement construct. Coding regions are indicated by filled boxes, untranslated regions by unfilled boxes, and introns by bent lines. Regions encoding the glutaminase and synthase domains are shown in blue and green, respectively. The position of the *pabB4* mutation is indicated (coordinates from v3.0 genome assembly, Lang *et al.* 2018). In the *∆adcs::mScarlet* knockouts lines, the coding region is replaced with DNA encoding a nuclear-localized mScarlet fluorescent protein and a *35S*:*aacC1* gene cassette to confer resistance to gentamicin flanked by *loxP* sites (orange triangles). The pink triangles indicate the positions targeted by the two CRISPR guide RNAs. **(B)** *Ssp*I-digested PCR products amplified from the *pabB4* mutant and Gransden WT. The mutation creates an additional cut site within a 343 base pair fragment resulting in 285 and 59 bp fragments. **(C)** Amino-acid alignment of a region in the glutaminase domain from plant, fungal, and bacterial sequences. A red arrow indicates Serine-302 and red bars indicate the histidine and glutamic acid active-site residues **(D–F)** Growth of strains on different media for 21 days: BCDAT minimal medium (D), BCDAT medium supplemented with 3 µM PABA (E), and BCDAT medium supplemented with 3 µM PABA and 50 µg/ml gentamicin (F). The *∆adcs::mScarlet* knockout lines (#10, 14, and 16), *pabB4,* and WT (Reute) are as labeled. **(G–H)** Confocal micrographs showing mScarlet fluorescence in the top panels and merged images of mScarlet (magenta), chlorophyll A (green), and calcofluor-white (blue) in the lower panels for the *∆adcs::mScarlet*#10 line (G) and Reute WT (H).

## Description

The haploid-dominant life cycle of mosses poses a challenge to genetically mapping infertile mutants. Recently, a method to circumvent these difficulties was developed that uses protoplast fusion of mutant and wild-type protoplasts to produce fertile somatic hybrids that produce segregating sporelings upon selfing (Moody *et al.* 2018). All known methods for producing somatic hybrids require the parent strains to either have complementing auxotrophic mutations or distinct antibiotic resistances (Grimsley *et al.* 1977; Cove *et al.* 2009b). Here we describe a strain that could be used as a universal fusion partner for mutants that contain neither type of marker, one that combines recessive auxotrophy with dominant antibiotic resistance.

Among the most commonly used auxotrophic mutants of the moss *Physcomitrium patens* (Hedw.) Mitt. (previously *Physcomitrella patens*) are those that require *p*-Aminobenzoate (PABA) for growth and which fall into two complementation groups, *pabA* and *pabB* (Ashton and Cove 1977; Grimsley *et al.* 1977; Ashton *et al.* 1979). PABA, along with pterin and glutamate moieties, is essential in the production of folates (Vitamin B_9_) which, in turn, are essential cofactors for one-carbon transfer reactions in the synthesis of various compounds such as methionine, purines, and thymidylates (reviewed in Hanson and Roje 2001). PABA is synthesized from chorismate and glutamine in three steps. The first two steps, glutamine hydrolysis and adding the resulting amino group to chorismate, are catalyzed by the Aminodeoxychorismate Synthase (ADCS) enzyme comprised of a single bifunctional protein in most plants and fungi and by separate glutaminase and synthase subunits in most bacteria (Basset *et al.* 2004a). (Note that the *P. patens* *pabA* and *pabB* complementation groups were named independently from the bacterial PabA and PabB enzyme subunits.) The 4-amino-4-deoxychorismate (ADC) product is converted to PABA by the ADC Lyase enzyme (Basset *et al.* 2004b).

In *P. patens*, ADCS is encoded by a single gene, *Pp3c26_2070*, whereas three genes (*Pp3c2_23040*, *Pp3c4_31240*, and *Pp3c7_15160*) appear to encode ADC lyase enzymes. We sequenced the *Pp3c26_2070* locus from the *pabB4* mutant and found a single mutation in the seventh exon that results in an asparagine substitution at a highly conserved serine residue (S302N) in the glutaminase domain immediately adjacent to the His-299 and Glu-301 active site residues and also creates an *Ssp*I restriction site (Fig. 1A–C). The corresponding serine in the bacterial glutaminase subunit was shown to form two critical hydrogen-bonds that link an Asp residue of the synthase subunit to a Thr residue of the glutaminase subunit; interactions between these three residues mediate the allosteric stimulation of glutaminase activity by chorismite binding to the synthase subunit (Semmelmann *et al.* 2019). Interestingly, the Asp residue is conserved in plant ADCS enzymes, but the Thr is not. It is not currently known whether chorismate binding also stimulates glutaminase activity in plant ADCS enzymes.

To confirm that *Pp3c26_2070* is required for PABA synthesis, we replaced its coding region in a wild-type strain’s genome with a cassette conferring resistance to gentamicin and a nuclear-localized mScarlet fluorescent-protein gene using CRISPR/Cas9-facilitated targeted gene replacement (Fig. 1A). Sixty-seven of seventy-two stable transformants assayed grew only on media supplemented with PABA, and three lines with clean gene replacements were selected based on PCR genotyping (*∆adcs::mScarlet*; Fig. 1D–1F). By comparison, the non-null *pabB4* mutant grew very slowly without added PABA (Fig. 1D and 1E). Weak fluorescent signal from mScarlet could be detected above background chloroplast-derived autofluorescence (Fig. 1G versus 1H).

The *∆adcs::mScarlet* mutant is potentially a useful genetic tool. It was designed to extend the mutant mapping system developed by Moody *et al.* (2018) to mutants whose background lack an antibiotic resistance that could be selected. After fusion with *∆adcs::mScarlet*, only bi-parental hybrids would be able to grow on minimal media supplemented with gentamicin—the mutant’s genome would provide a functional *ADCS* gene and *∆adcs::mScarlet*’s genome would confer gentamicin-resistance. Such a universal hybridization partner might also allow production of allopolyploid lines through fusion with protoplasts from other moss species, most of which lack established transformation protocols. Auxotrophic mutants may also prove essential in the development of stably maintained shuttle vectors in moss. Unlike other plants, *P. patens* can maintain plasmid DNA extrachromosomally as long as selection is applied (Ashton *et al.* 2000; Murén *et al.* 2009). Vectors that complement auxotrophic mutations may be superior to those that confer antibiotic resistance because selection can be maintained even in cells not in direct contact with the substrate (Ulfstedt *et al.* 2017).

## Methods

Moss propagation and transformation were carried out as described previously (Cove *et al.* 2009a; Cove *et al.* 2009c). The *Pp3c26_2070* gene was amplified and sequenced in four segments using the indicated primers (Primer Table) from the *pabB4* mutant (kindly provided by Neil Ashton, University of Regina). To confirm that the G-to-A mutation in the seventh exon was unique to the *pabB4* genome, the region including the mutation was amplified from *pabB4* (Gransden background) and the ‘Gransden 2004’ wild type and digested with *Ssp*I. The *Ssp*I site created by the G-to-A mutation was only present in the *pabB4* product.

The *∆adcs::mScarlet* construct, pMP1907, was created by ligating in *Spe*I–*Swa*I and *Sph*I–*Sal*I fragments with the downstream and upstream homology arms, respectively, into the pMP1119 vector. pMP1119 was created from pBNRF (Thelander *et al.* 2007) by 1) digestion with *Bgl*II and *Not*I followed by polishing with T4 DNA polymerase and re-ligation, 2) removal of the *35S:nptII* transgene by *Eco*RI digestion and re-ligation, and 3) insertion of the *35S:aacC1* transgene as a *Kpn*I–*Sac*I fragment after amplifying from pYL-TAP-Nt (Rubio *et al.* 2005). The *Pisum* *rbcS-E9* terminator sequence was inserted as a *Kpn*I fragment upstream from the *35S:aacC1* transgene. NLS-mScarlet (Bindels *et al.* 2017) was amplified and subcloned into pCR Blunt (ThermoFisher) then inserted as an *Eco*RI fragment into the *Mfe*I site. The plasmid to express *SpCas9* and guide RNAs designed to target near the start- and stop codons (pMP1957) was generated using oligos and pMK-Cas9-gate according to published protocols (Mallett *et al.* 2019). Fifteen µg of both pMP1907 and pMP1957 plasmids were transformed into the Reute 2016 strain (Hiss *et al.* 2017). After regeneration on PRMB medium containing 3 µM PABA, four-day-old transformed protoplasts were selected on BCDAT medium containing 3 µM PABA, 100 mg/l gentamicin, and 20 mg/l G418 for one week. Transformants were picked to BCDAT+PABA medium, then 10 days later a small clump of each was transferred to BCDAT+PABA+gentamicin medium to identify stable transformants. We later discovered that expression of *aacC1* confers resistance to both gentamicin and G418 (but not to 100 mg/l kanamycin), however the high rate of stable integration likely reflects the high rate of co-transformation despite no selection for pMP1957 uptake. The presence of proper 5′ and 3′ integration products and the absence of *SpCas9* and *ADCS* genes were confirmed by PCR (Primer Table).

mScarlet, calcofluor-white, and chlorophyll fluorescent signals were imaged using a Zeiss LSM 880 microscope using 561, 405, and 633 nm excitation and 580–605, 410–501, and 647–721 nm detection windows, respectively.

## Reagents

**Table:** 

**Primer Table**		
**Name**	**Sequence (5′ to 3′)**	**Purpose**
P35S-KpnF	atcggtaccAACATGGTGGAGCACGAC	Subcloning *35S:aacC1*
T35S-SacR	tcggagctcCTGGATTTTGGTTTTAGGAATTAGA	Subcloning *35S:aacC1*
SV40FP-XbaF	tctagaATGGCTCCAAAGAAGAAGAGAAAGGTCGCTGTGAGCAAGGGCGAGGA	Subcloning *NLS-mScarlet*
mCherry-xbaR	tactctagaTTACTTGTACAGCTCGTCCATGC	Subcloning *NLS-mScarlet*
Te9-KpnF	TCCggtaccGTTCGAGTATTATGGCATTGGG	Subcloning *rbcS-E9*
Te9-KpnR	GTTgGtACcATTGGCAAGTCATAAAATGCATT	Subcloning *rbcS-E9*
ADCS5-SphF	CAAgcATGCTTTTTTTCAAAGCAAATTTG	Subcloning 5′ targetting arm
ADCS5-SalR	TGCgtCGACCTCAAGCTCCATTTTCAGACC	Subcloning 5′ targetting arm
ADCS-3SpeF	GAAacTAGTGTGGCTTTACCTTAGTCTCCTC	Subcloning 3′ targetting arm
ADCS-3R	AACACCTTCACTTATATGCCTCCA	Subcloning 3′ targetting arm
ADCS-5-crF	ccatGCACCTGGAGATGACTCAGA	CRISPR protospacer
ADCS-5-crR	aaacTCTGAGTCATCTCCAGGTGC	CRISPR protospacer
ADCS-3-crF	ccatACACCACCTCCAGCAGTCAA	CRISPR protospacer
ADCS-3-crR	aaacTTGACTGCTGGAGGTGGTGT	CRISPR protospacer
ADCS-5uF	GGTCTGAAAATGGAGCTTGAGGT	Sequencing
ADCS-4iR	AGGAGAAGAAGGAGCAAAGCAGA	Sequencing
ADCS-4iF	CGGTCGTTTAAGGTATAATTTCTCCA	Sequencing, *pabB4* genotyping
ADCS-8eR	ATCAGAACATGGCTTGAATCGTC	Sequencing, *pabB4* genotyping
ADCS-8eF	GATCTTACGAAGTGCCTGCATGA	Sequencing
ADCS-12eR	TAGAGAGCCATTCGACTTGGAAAC	Sequencing
ADCS-12eF	ATTCGTTTAATCACGGCCAGAAC	Sequencing
ADCS-3uR	ATCCCCTGATGGAACTACGTGAA	Sequencing
Ubi-tataF2	CGATGCTCACCCTGTTGTTTGG	∆*adcs* genotyping (*Cas9*)
Cas9-R	TTGATCATGGAGGCGGAGAGTG	∆*adcs* genotyping (*Cas9*)
ADCS-genoF	GGATAGAGCCCCACAAAGCCA	∆*adcs* genotyping (5′)
NLS-genoR	ACCTTTCTCTTCTTCTTTGGAGCCA	∆*adcs* genotyping (5′)
Tcamv-genoF	CCTATAGGGTTTCGCTCATGTGTTG	∆*adcs* genotyping (3′)
ADCS-genoR	CCAATAAGTCCTACCAAATAAACGCCT	∆*adcs* genotyping (5′)
ADCS-e6F	ATGCTCCTGGGGTTGATTGCT	∆*adcs* genotyping (WT)
ADCS-e11R	CACAAAAGTGGAAGGGCAGGC	∆*adcs* genotyping (WT)
